# High Diversity of the Saliva Microbiome in Batwa Pygmies

**DOI:** 10.1371/journal.pone.0023352

**Published:** 2011-08-16

**Authors:** Ivan Nasidze, Jing Li, Roland Schroeder, Jean L. Creasey, Mingkun Li, Mark Stoneking

**Affiliations:** 1 Max Planck Institute for Evolutionary Anthropology, Leipzig, Germany; 2 D.D.S., Nevada City, California, United States of America; Universidad Miguel Hernandez, Spain

## Abstract

We describe the saliva microbiome diversity in Batwa Pygmies, a former hunter-gatherer group from Uganda, using next-generation sequencing of partial 16S rRNA sequences. Microbial community diversity in the Batwa is significantly higher than in agricultural groups from Sierra Leone and the Democratic Republic of Congo. We found 40 microbial genera in the Batwa, which have previously not been described in the human oral cavity. The distinctive composition of the salvia microbiome of the Batwa may have been influenced by their recent different lifestyle and diet.

## Introduction

The Batwa Pygmies, also known as Twa, are believed to be the original inhabitants of the equatorial forests of the Great Lakes region of Central Africa [1]. They live in southwestern Uganda, northern and southern Rwanda and in many areas of the Kivu province of the Democratic Republic of the Congo (DRC).

Traditionally, the Batwa have been semi-nomadic hunter-gatherers. However, relatively recent human activities, such as clearing of the forests for agriculture and creation of conservation areas, have pushed the Batwa from their traditional homeland and as a consequence their lifstyle has changed dramatically. Most Batwa now live on the borders of forest and agricultural areas but still use the forest on a daily basis. Batwa groups are small, rarely exceeding 50 people, and are often based around members of a particular clan.

An interersting feature of the Batwa is that they differ significantly from neighboring Bantu agriculturalists in having fewer caries lesions and reduced tooth loss [2]. Differences in diet and lifestyle provide the most likely explanation for the greater prevalence of caries lesions and tooth loss among the Bantu than among the Batwa. The Batwa have less access to highly cariogenic, refined carbohydrates than do the Bantu. In addition, because of their hunter-gatherer lifestyle, the diet of the Batwa tends to be higher in animal protein than that of the Bantu, and this would also contribute to a lower caries rate [1].

It is also possible that the oral microbiome of the Batwa may either influence, or be influenced by, the lower prevalence of caries.To investigate this further, we analyze here the saliva microbiome diversity of the Batwa in comparison with agricultural groups from similar enviroments in Africa, in order to address the following questions: 1) how different is the Batwa saliva microbiome from that of African agriculturalists; and 2) is the low level of dental caries in the Batwa associated with particular microbial taxa?

## Materials and Methods

### Ethics statement

All participants gave written informed consent. The protocol was in accordance with the Helsinki Declaration, and was approved by the Ethics Commission of the University of Leipzig Medical Faculty.

### Samples and DNA extraction

**Figure 1:**
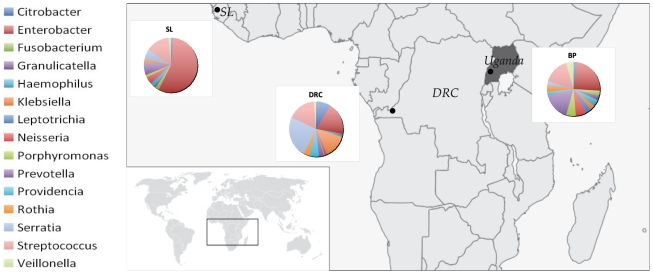
A map of sampling locations and piecharts showing the frequencies of the 15 most common microbial genera in these groups.

Saliva samples were collected from: 39 Batwa from the Mpungo, Mukongoro, Kitariro, Nyakatare, Bikuto commuinities of Buhoma, Uganda: 20 individuals from Kinshasa, Democratic Republic of Congo (DRC); and 13 individuals from Freetown, Sierra Leone (SL). (Figure 1). DNA was extracted as described previously [3].

### PCR amplification of the microbial 16S rRNA gene

We amplified a region of the microbial 16S rRNA gene containing variable segments V1 and V2, which were previously shown to be more informative than other parts of the 16S rRNA gene in terms of the number of phylotypes detected [4]. We used the forward primer for V1 and the reverse primer for V2 [4], which together amplify a ∼350 bp PCR product containing V1 and V2.

### Sequencing on the Genome SequencerFLX platform

The PCR products were processed for parallel-tagged sequencing on the Genome Sequencer FLX platform, as described previously [5], [6]. Briefly, sample-specific barcode sequences were ligated to the PCR products, and DNA concentrations were assessed on a Mx3005P™ (Stratagene). Samples were then pooled in equimolar ratios to a total DNA amount of 440 ng. The pooled library was subsequently amplified in PCR-mixture-in-oil emulsions and sequenced on one lane of a 4-lane PicoTiterPlate on a Genome Sequencer FLX/454 Life Sciences sequencer (Branford CT), according to the manufacturer's protocol. The negative control was sequenced on an individual lane.

### Data analysis

The initial sequence reads were filtered to remove artifactual sequence reads (i.e., reads containing two or more different tags, no tags, primers in the middle of sequence reads, or without a primer sequence). The filtered sequences were then searched against the *Ribosomal Database Project II* (RDPII) database [7], using the online program SEQMATCH (http://rdp.cme.msu.edu/seqmatch/seqmatch_intro.jsp) and a threshold setting of 90%, to assign a genus to each sequence. Diversity statistics and apportionment of variation based on the frequency distribution of genera within and between individuals were calculated with Arlequin 3.1 [8], while pairwise correlation analysis and principal component analysis (PCA) were carried out using STATISTICA 6.1 (StatSoft, Inc.) [9]. Mann-Whitney U tests [10] were used to compare distributions of correlation coefficients. Rarefaction analysis was carried out using the Resampling Rarefaction 1.3 software (http://www.uga.edu/~strata/software/index.html ). UniFrac analysis [11] was used to compare microbial community diversity in a phylogenetic context.

Comparative data on the salivia microbiome diversity in two agricultural groups, from the Democratic Republic of Congo (DRC) and Sierra Leone (SL), came from another study (J. Li, I. Nasidze, and M. Stoneking, unpublished data).

## Results

A total of 29728 sequence reads were obtained from the Genome Sequencer FLX. After filtering and removing sequence reads less than 200 bp, 24358 sequences remained (Table 1). Sequences shorter than 200 bp give potentially unreliable results when compared to the RDPII database and were therefore excluded from further analysis; more than 81.9% of the sequence reads were at least 200 bp (Figure S1).

**Table 1:** Number of sequence reads, detected genera and AMOVA.

	No. of	Unknown	Total no. of	No. of genera per individual	Variance between	Variance within
	sequences	%	genera	min - mean - max	individuals (%)	individuals (%)
Batwa Pygmy	24358	0.02	127	11 - 30 - 61	16.27	83.73
DRC*	5353	0.7	54	8 - 13 - 22	32.12	67.88
SL*	17820	0.7	71	12 - 23 - 37	30.57	69.43

*data from J. Li, I. Nasidze and M. Stoneking (unpublished data).

**Figure 2:**
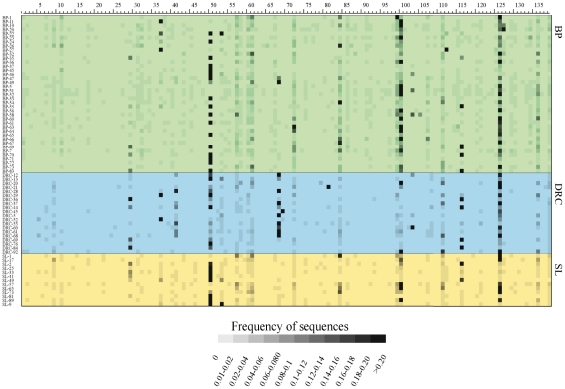
Heat plot of the abundance of each bacterial genus in each individual, based on the partial 16S rRNA sequences. Each numbered column corresponds to a genus, with the genus name for each number indicated in Table S1. Each row is an individual saliva sample. The abundance of each genus is indicated by the grayscale value, according to the scale at the bottom of the plot. BP – Batwa Pygmy; DRC – group from the Democratic Republic of the Congo; SL – group from Sierra Leone.

**Figure 3:**
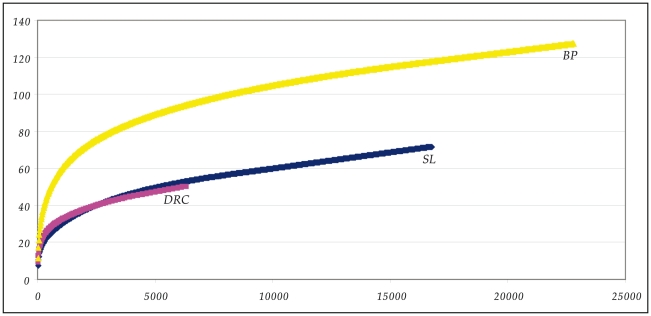
Rarefaction analysis of the number of bacterial genera detected in the three human groups as a function of the number of reads. The data points represent averages of 1000 randomized resamplings without replacement.

The results of comparing these sequences to the RDPII database are provided in Table S1 and illustrated in the heat plot in Figure 2. Altogether, 99.8% of the sequences matched a previously-identified genus, while 0.2% were unknown (did not match any sequence in the database above the 90% threshold value). In the following analyses we focus only on those sequences that matched a known genus in the RDPII database. A total of 127genera were detected in the 39 Batwa, compared to 71 genera in the SL group and 54 genera in the DRC group (Table 1); overall, 143 microbial genera were detected (Table S2). Although many more genera were detected in the Batwa than in the other two groups, there were also many more sequence reads obtained from the Batwa than from the other two groups, as well as more reads obtained for the SL group than the DRC group (Table 1). In order to test whether or not the differences in the number of detected genera are due to the diffferences in number of sequence reads, we carried out a rarefaction analysis (Figure 3). The rarefaction curves for the SL and DRC groups overlap, indicating that the different numbers of genera detected for these two groups can be explained by the difference in number of sequence reads. However, the curve for the Batwa increases much more rapidly compared with the other two groups, indicating that for similar numbers of sequence reads, many more genera are detected in the Batwa than in the other two groups. Thus, the Batwa are distinguised from the other two groups in having many more genera in their saliva microbiome.

A list of the top fifteen most frequent microbial genera found in these groups and their distribution is shown in Figure 1. These genera account for 81.9–89.4% of the total number of reads obtained for each group.

**Figure 4:**
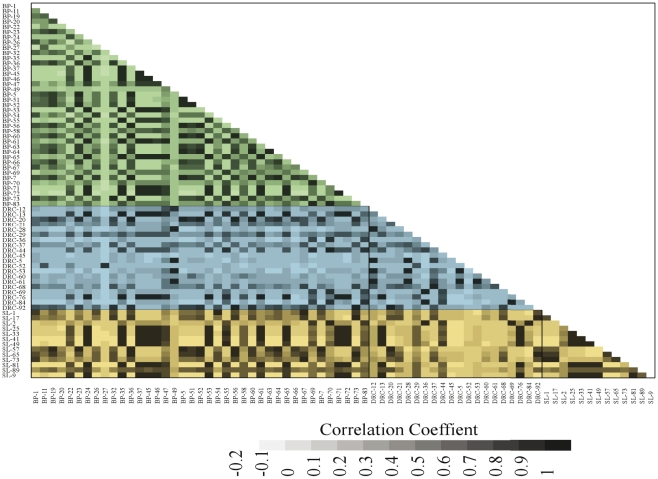
Pairwise correlation matrix between data sets, both within groups and between groups. The correlation coefficient values are indicated by the grayscale value, according to the scale at the bottom of the matrix.

Given the overall differences in the genera detected among the three groups, how strongly correlated are the saliva microbiome compositions of different individuals within and between groups? To address this question, we calculated correlation coefficients for the distribution of genera detected between each pair of individuals, both within and between groups (Figure 4). In general, with only a few exceptions, there are positive correlations within each group. The average correlation coefficient within the Batwa and within the SL group are similar (0.40 and 0.42, respectively), while it is much lower within the DRC group (average correlation coefficient = 0.26). The average correlation is also higher between the Batwa and the SL (0.42) than between the Batwa and DRC or between the SL and DRC.

**Figure 5:**
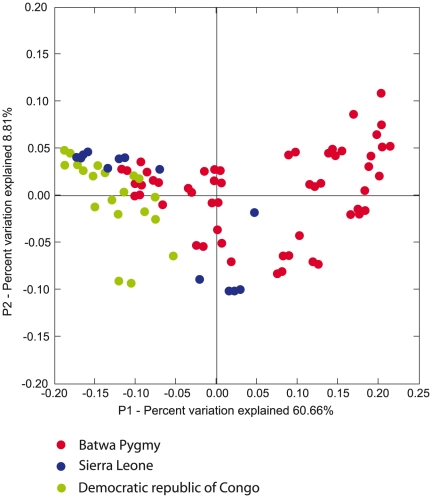
Principal coordinate plot based on genera frequencies showing relationships among the oral microbiome composition in Batwa Pygmy and human groups from DRC and SL. Color coding for each group is shown at the bottom of the figure.

We also carried out an analysis of molecular variance (AMOVA) in order to investigate how much of the total variation in the saliva microbiome is due to differences within vs. among individuals from each group. The results indicate that the SL and DRC groups have very similar apportionments of variation, but the Batwa have much less differentiation between individuals, with 16% of the variation between individuals, vs. 30–32% for the other two groups (Table 1). Thus, the Batwa are characterized by more overall diversity but more similarity among individuals in their saliva microbiome, compared to the other two groups. Pricipal component analysis (PCA; Figure 5) showed a quite distinct position of most of the Batwa, separate from individuals from SL and DRC. The latter two groups are not clearly separated from each other.

**Figure 6:**
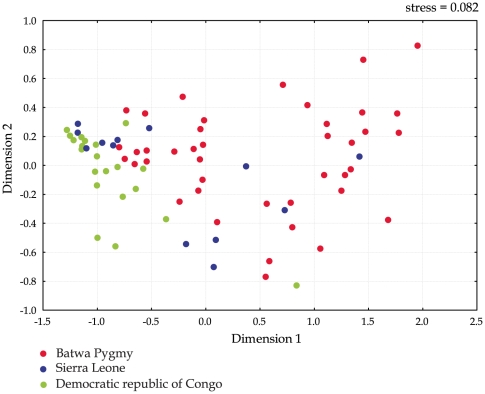
Multidimentrional scaling plot based on UniFrac distances, showing relationships among individuals based on saliva microbiome composition. Color coding for each group is the same as for Figure 5.

In order to test whether the patterns observed in the PCA plot are also seen at the sequence level, and not just from inferred genera, we also carried out UniFrac analysis and multidimenional scaling (MDS) based on UniFrac distances. The resulting MDS plot is highly consistent with the patterns observed in the PCA plot (Figure 6).

## Discussion

To gain further insights into the influence of lifestyle and diet on the human saliva microbiome, we compared the saliva microbiomes of Batwa Pygmies (who were, until recently, traditional hunter-gatherers) with two farming groups, from Sierra Leone and the Democratic Republic of the Congo.We found significantly higher diversity in the saliva microbiome of the Batwa (127 different genera) compared with the groups from DRC (54 different genera) and SL (71 different genera). Rarefaction analysis (Figure 3) indicated that this difference between the Batwa and the other groups is not due to the higher number of sequence reads for the Batwa, but instead reflects a true excess of the number of microbial genera in the Batwa. One potential factor could be the protein-rich diet of the Batwa, as more than 49% of their diet consists of hunted animal meat [2]. However, in the last two decades an agricultural diet has become more prevalent in the Batwa.

We found 59 microbial genera that are unique to the Batwa (Table S2). Although most of them are present in very low frequency, some are present in higher numbers. For example, *Cloacibacterium*, found at low frequency in several Batwa, was discovered recently but in a different enviroment, namely in municipal wastewater [12] and freshwater lake sediments [13].

We compared the list of microbial genera found in Batwa with the Human Oral Microbiome Database [14] and with our previous study of the saliva microbiome from worldwide populations [15], and found that almost a third of the microbial genera detected in the Batwa (40 genera) have not been described in the human oral cavity before (Table S2). This finding suggests that the human oral cavity harbors a much more diverse microbial community than described so far, and illustrates the importance of analyzing the oral microbiome in diverse human populations.

Although groups from SL and DRC are geographically distant (figure 1), they show a higher degree of similarity to each other than with the Batwa. The average genetic distance (Fst value) between these groups is lower (0.183) than with the Batwa (0.226 and 0.242 respectively). This patterns are obvious also from MDS and PC plots. This observation suggests that similar lifstyles and diet can lead to more similar oral microbiomes, although larger scale studies that include more groups from different parts of the world are needed to further investigate this.

One of the interesting features of the Batwa is a very low incidence of dental caries, compared with other neighboring groups. A key environmental factor influencing pathogenicity of the oral biofilms that colonize the hard tissues of the human mouth is pH [16]. Alkali generation, particularly through ammonia production from arginine and urea, plays a major role in pH homeostasis in oral biofilms and may protect from the initiation and progression of dental caries [16]. There are two major substrates for alkali production by oral biofilms colonizing the teeth, urea and arginine; urea is present in saliva and is rapidly hydrolyzed to ammonia and CO_2_ by bacterial ureases which are produced by a particular subset of oral bacteria, including *Actinomyces naeslundii* and oral *Haemophilus* [16]. The frequency of reads assigned to *Actinomyces* does not vary significantly between the Batwa and the other two human groups, but for *Haemophilus*, the frequency of reads is significantly higher in the Batwa (0.034) than in the other two groups (0.021 in SL and 0.009 in the DRC; chi-square test for equal frequencies, p<0.0001). Moreover, *Haemophilus* was identified in almost every Batwa individual (Table S2). We speculate that this significantly higher frequency of *Haemophilus* in the saliva microbiome might play a role in the reduced dental caries in the Batwa.

In conclusion, the Batwa exhibit higher diversity in their saliva microbiome composition. A number of unique genera are found only in the Batwa, and the lifestyle and diet of the Batwa may play a role in their distinctive saliva microbiome. Our results also highlight the importance of studying the microbiomes of non-traditional groups, in order to fully encompass the diversity of the human microbiome.

## Supporting Information

Figure S1 **Length distribution of the reads of Batwa Pigmy.**
(EPS)

Table S1 Count of detected microbial genera in Batwa Pygmy individuals.
(XLS)

Table S2 Count of sequence reads assigned to each detected microbial genus in Batwa and groups from the Democratic Republic of the Congo (DRC) and Sierra Leone (SL).
(XLS)
